# Moderating effect of pain sensitivity on dental anxiety: a randomized controlled cognitive-behavioral intervention trial

**DOI:** 10.1007/s10865-026-00691-1

**Published:** 2026-07-01

**Authors:** Eugene M. Dunne, Elizabeth Konneker, Huaqing Zhao, Amid I. Ismail, Marisol Tellez

**Affiliations:** 1 Maurice H. Kornberg School of Dentistry, Temple University, 3223 N. Broad Street, Room L217, Philadelphia, PA 19140 USA; 2 Center for Biostatistics and Epidemiology, Department of Biomedical Education and Data Science, Lewis Katz School of Medicine, Temple University, Philadelphia, PA USA

**Keywords:** Dental anxiety, Pain sensitivity, Cognitive behavioral therapy

## Abstract

Pain sensitivity is considered a stable characteristic that can influence the development of situational fears. It has been related to dental anxiety and expectation of pain before dental procedures. The objective of this analysis was to examine whether baseline pain sensitivity moderated the efficacy of an online cognitive-behavioral therapy (CBT) intervention for dental anxiety relative to an active control condition. Participants with high dental anxiety were recruited from a dental school clinic in the northeastern United States to participate in a randomized controlled trial. Patients were randomized to receive an online CBT intervention or a time- and attention-matched control intervention. Changes in dental anxiety were assessed using the Modified Dental Anxiety Scale (MDAS). Baseline pain sensitivity was measured by the Pain Sensitivity Index (PSI), which assesses the fearful appraisal of pain and expected consequences of pain. Participants completed questionnaires at baseline, and at 1- and 3-month follow ups. Linear mixed-effects model analyses using mean-centered interaction terms examined whether baseline levels of pain sensitivity moderated the treatment effect on dental anxiety. A total of 499 participants (70.6% female, 62% Black, *Mean* age = 49) were enrolled in the clinical trial and randomized to the CBT intervention (*n* = 329) or control condition (*n* = 170). Baseline pain sensitivity ratings between intervention group (*Mean* = 67.4, *SD* = 22.3) and control group (*Mean* = 67.6, *SD* = 24.2 did not significantly differ (*t* = − 0.10, *p* = 0.92). Baseline pain sensitivity significantly moderated the effect of the online CBT intervention on dental anxiety, such that participants with low pain sensitivity were significantly more likely to see reductions in dental anxiety when compared to those with high pain sensitivity, (*F*(1, 633) = 5.91, *p* = 0.015). Participants with lower pain sensitivity experienced the greatest benefit in terms of reduction in dental anxiety following an online CBT intervention. Individuals with high pain sensitivity may require more targeted interventions or higher intervention dose to achieve adequate reduction in dental anxiety.

## Introduction

Fear and anxiety related to dental care are common, with recent prevalence estimates of 15% among adult populations (Silveira et al., 2021). Individuals with dental anxiety are more likely to delay or avoid dental appointments, including preventative care visits (Armfield & Ketting, 2015). Persistent avoidance of dental care can lead to poor oral health and increase the likelihood of more invasive, painful, and costly dental procedures. The cycle of fear and avoidance is therefore reinforced, as the feared situation (i.e., painful dental treatment) is more likely to be experienced (Seligman et al., 2017).

The experience of pain is highly subjective and variable, both between individuals and within individuals across time (Nielsen et al., 2009). However, pain sensitivity is considered a stable characteristic that can influence the development of dental-related fears (Gross, 1992). Sensitivity to painful stimuli is one factor that impacts the degree to which an individual will experience and report pain, and how they respond to pain relief intervention. Individuals with higher pain sensitivity are likely to focus their cognitive resources and sensory receptors towards the detection of pain. This pain hypervigilance increases the likelihood of reporting higher pain severity (Herbert et al., 2014). Prior studies have established that pain sensitivity is related to dental anxiety and the expectation of pain before dental procedures (Klages et al., 2004) yet is a distinct construct from dental phobia and health concerns (Gross, 1992). Thus, pain sensitivity may play an important role in the fear and avoidance cycle of dental anxiety.

Psychological interventions can be used to break this cycle of dental fear and avoidance, particularly cognitive behavioral therapy (CBT) interventions using an exposure-based paradigm (Tellez et al., 2015). Cognitive behavioral therapy is a structured form of psychotherapy that is goal-oriented and guides the patient towards a better understanding of their thoughts (i.e., cognitions), emotions, and behaviors (Beck, 2020). CBT is an evidence-based treatment and is considered the gold-standard, front-line approach to treating depression, anxiety, and other mental health diagnoses (Butler et al., 2006). CBT for anxiety typically includes an exposure and response prevention paradigm, where the patient generates a list of feared stimuli (i.e., fear hierarchy) and is gradually exposed to each level while learning coping strategies to prevent avoidance behaviors. Several adaptations of CBT have been explored for addressing dental anxiety, including brief and computerized programs (Tellez et al., 2015; Heyman et al., 2024), with varying degrees of efficacy. Importantly, psychological and personality characteristics (e.g., pain sensitivity) may impact the degree to which an individual will engage with and benefit from a CBT intervention with exposures to feared stimuli. The present study sought to explore this research question with a sample of adult participants with dental anxiety who were enrolled in a randomized controlled trial evaluating a CBT intervention for dental anxiety (Tellez et al., 2025). The objective of this secondary analysis was to examine whether baseline pain sensitivity moderated the efficacy of the online exposure-based CBT intervention for dental anxiety relative to an active control condition.

## Methods

### Participants

**Fig. 1:**
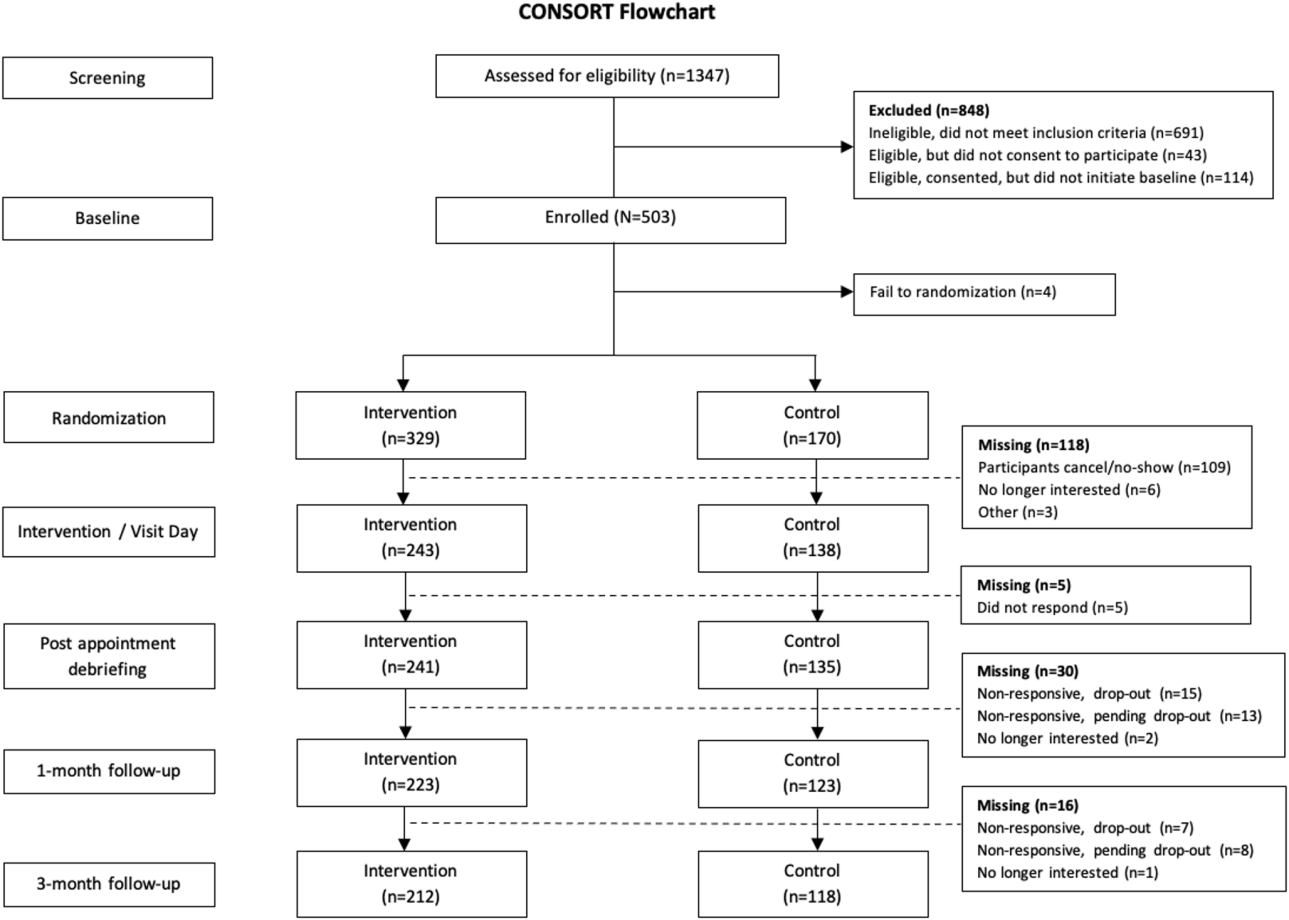
CONSORT flow chart. *Note* participants were considered as “drop out” if they missed two consecutive assessments

Participants with upcoming appointments were recruited from multiple clinics at an urban dental school in the northeast United States between 2019 and 2023. Participants were eligible if they were between 18 and 75 years of age and self-reported dental anxiety on a screening measure. Dental anxiety was determined by scoring 19 or higher on the Modified Dental Anxiety Scale (MDAS; Humphris et al., 1995) or endorsing “extreme anxiety” on at least two of the five items. Eligible participants provided informed consent and completed a series of initial baseline assessments. Next, participants were randomized to one of three parallel conditions: CBT with a dental-trained therapy aid (e.g., dental assistant), CBT with a psychology-trained therapy aid (e.g., psychology post-doc fellow), or a time- and attention-matched control group. Randomization was determined by a computer-generated number sequence, secured and restricted to one staff member, and communicated to the research staff immediately following baseline assessment. Figure 1 illustrates the CONSORT flow chart of participants from screening through 3-month follow-up. Participants attended the intervention or control condition visit two hours before their scheduled dental appointment. Following their dental appointment, participants were contacted within 24 h for a brief immediate follow-up survey. Lastly, participants completed follow-up assessments at 1-month, 3-months, 6-months, and 12-months. Assessments were completed online or by phone, and participants indicated whether they preferred to receive reminders about assessments by phone or email. Participants earned up to $175 for completing all study visits and assessments. The study procedures were approved and reviewed annually by an institutional review board (IRB) and additional oversight was provided through an independent medical monitoring committee. There were no harms or adverse events reported. A detailed description of the RCT procedures was previously published (Tellez et al., 2025).

Previous studies have established acceptability and efficacy for the online CBT intervention for managing dental anxiety (Tellez et al., 2015). While previous studies used psychology-trained staff to administer the intervention, the current randomized controlled trial (ClinicalTrials.gov ID: NCT03680755) was designed to determine whether the intervention could be similarly administered by dental-trained staff (i.e., non-inferiority). Results of the primary analysis indicated that both groups benefited equally from the intervention program (Tellez et al., 2025). As such, the current secondary analysis combines the two active arms (i.e., dental aid and psychology aid) to explore the moderating effects of pain sensitivity across all participants who received either the intervention or control group.

The online CBT intervention was delivered through a desktop computer in a private office at the dental school. The therapy aid met with the participant, oriented them to the computerized program, and was available in the room to answer any questions or concerns. Though not measured explicitly, participants rarely interacted with therapy aids while engaged with the intervention content, with the occasional exception of addressing technology issues (e.g., screen/frame frozen). The intervention lasted approximately one hour and consisted of a series of educational, motivational, and exposure videos. Participants were first educated on the impact of dental anxiety and reasons to work on addressing dental anxiety. Next, participants selected their three most feared dental procedures from a list of options, including injection, deep cleaning, root canal, tooth extraction, and cavity filling. The psychology and dental therapy aids received two hours of training related to dental anxiety and cognitive behavioral interventions. The time- and attention-matched control condition consisted of a 60-minute nature video. Participants in the control condition were invited to return to the dental school to receive the active intervention (i.e., online CBT program) after completion of all study follow-up assessments.

### Measures

#### Participant characteristics

Demographic information was collected at baseline assessment, including age, sex, gender, racial and ethnic background, marital status, education, and employment.

#### Dental anxiety

The Modified Dental Anxiety Scale (MDAS) is a five-item measure which assesses anxiety related to specific events prior to and during dental appointments. Specifically, it asks participants to rate their level of anxiety if they had a dental appointment the following day, in the waiting room, having a tooth drilled, getting a deep cleaning, or receiving an injection. Participants respond using a five-point Likert-type scale ranging from “not anxious” to “extremely anxious.” Scores range from 5 to 25, with scores of 19 and above indicating significant dental anxiety. The MDAS is among the most used measures of dental anxiety and has demonstrated strong psychometric properties, including reliability and validity (Humphris et al., 1995, 2009).

#### Pain sensitivity

The Pain Sensitivity Index (PSI) is a 16-item self-report measure that assesses the fearful appraisal of pain and the expected physical, psychological, and social consequences of pain. Items are rated on a scale ranging from 0 (not at all) to 7 (very much) reflecting the degree to which the item applies to the respondent. Higher scores indicate higher levels of pain sensitivity. The scale has demonstrated high internal consistency in previous studies with dental patients (α = 0.92) (Klages et al., 2004).

### Data management and analyses

This proposed moderation analysis was a planned secondary research question of a clinical trial evaluating the efficacy of the online CBT program for dental anxiety. For these analyses, the two active CBT intervention groups (psychology-trained therapy aid and dental-trained therapy aid) were combined for comparison to the control condition. Descriptive statistics (e.g., count, percentage, mean, standard deviation) were used to define sample characteristics. Baseline group differences were explored using bivariate analyses (e.g., t-test, chi-square). Linear mixed-effects model analyses using mean-centered interaction terms examined primary intervention effects for group (intervention vs. control) and time (baseline, 1-month, and 3-month), and whether baseline levels of pain sensitivity moderated the treatment effect on dental anxiety. Using the mean-centered values, post-hoc analyses classified participants as “high” or “low” on the pain sensitivity index to illustrate the moderation effect on the intervention and control conditions. Data analyses were conducted using SPSS Version 28 (IBM Corp, Armonk, NY).

## Results

### Participant characteristics

Recruitment outreach calls were made to 7030 patients with upcoming appointments. Among these, 1374 participants expressed interest in the study and were assessed for eligibility. Patients were not enrolled in the study if they failed to meet inclusion criteria (e.g., did not meet minimal dental anxiety MDAS score), declined to participate after screening, or were lost to follow-up before initiating consent and baseline. Among patients who were eligible, there were no significant differences between patients who enrolled and those who declined or failed to enroll. A total of 499 participants were eligible and enrolled in the clinical trial.

Enrolled participants were randomized to the CBT intervention (*n* = 329) or control condition (*n* = 170). The majority of participants (70.6%) reported female gender, with 28.3% male. Regarding race, 62.4% of participants identified as Black or African American, while 26.8% reported White/Caucasian, and 4.6% were Asian. 11% of participants identified as Hispanic/Latino ethnicity. Mean participant age was 49 years (*SD* = 14.9). Bivariate analyses exploring baseline participant characteristics found no significant demographic differences between the intervention and control groups (Table 1; all *p* > 0.05).

**Table 1:** Baseline participant characteristics and demographic information

	Total *N =* 499	Online CBT *N =* 329	Control *N =* 170	*p*
*Race*				0.16
White or Caucasian	129 (26.8%)	90 (27.4%)	39 (23.6%)	
Black or African American	301 (62.4%)	191 (58.1%)	110 (66.7%)	
Asian	22 (4.6%)	16 (4.9%)	6 (3.6%)	
Others	30 (6.3%)	20 (6.1%)	10 (6.0%)	
*Ethnicity*				0.84
Hispanic/Latino	56 (11.4%)	36 (11.0%)	20 (11.8%)	
Non-Hispanic	434 (88.6%)	285 (86.6%)	149 (88.2%)	
*Gender*				0.54
Female	354 (70.9%)	228 (69.5%)	126 (73.5%)	
Male	140 (28.1%)	96 (29.3%)	44 (25.7%)	
Others	5 (1.0%)	4 (1.2%)	1 (0.6%)	
Age, Mean ± SD	49.0 ± 14.9	49.5 ± 14.9	48.0 ± 14.8	0.31
MDAS, Mean ± SD	19.5 ± 3.6	19.4 ± 3.8	19.8 ± 3.1	0.28

Not included in this table are data that were missing responses or participant refused to answer for race (*n* = 17) and ethnicity (*n* = 9)

At baseline, participants in the intervention group and control group reported similar levels of pain sensitivity, with non-significant differences (*t* = − 0.10, *p* = 0.92) between the intervention group (*Mean* = 67.4, *SD* = 22.3) and control group (*Mean* = 67.6, *SD* = 24.2). Participants in the intervention group reported a mean dental anxiety score of 19.4 (*SD* = 3.7) while those in the control group scored 19.8 (*SD* = 3.2), which was non-significant (*t* = − 1.09, *p* = 0.28).

### Moderation analysis

**Fig. 2:**
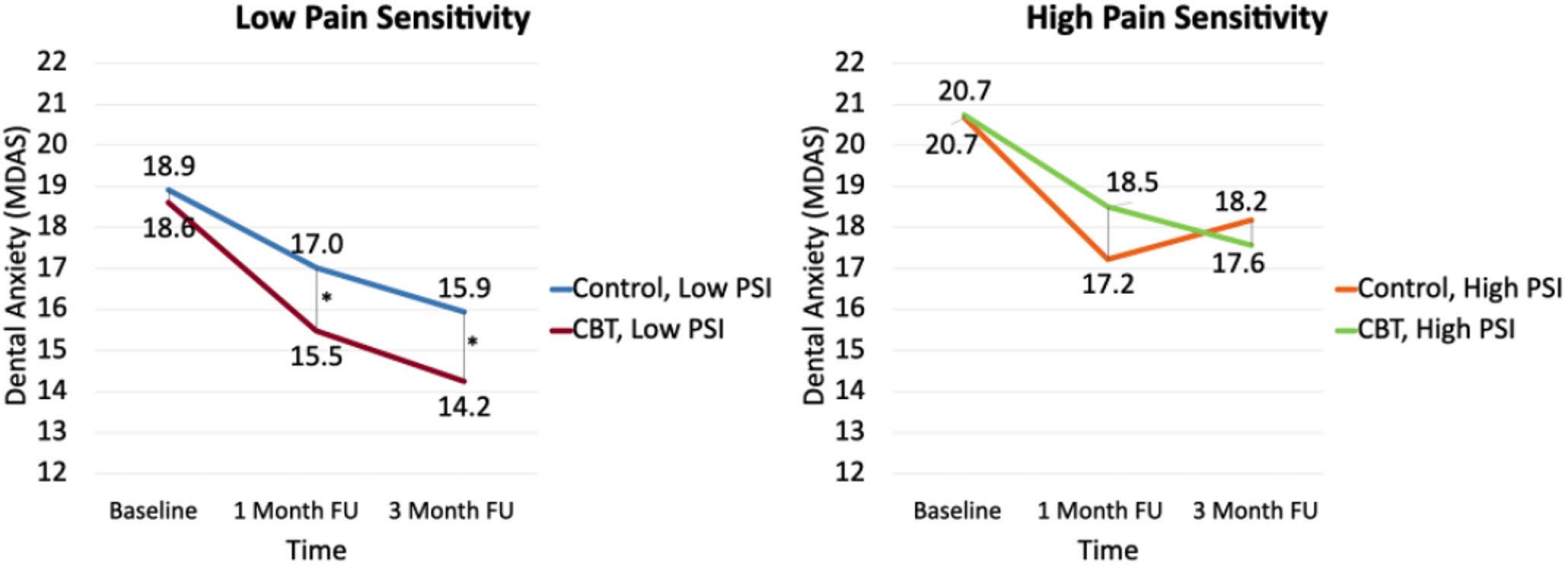
Linear mixed-effects model indicated significant effects for condition, *F*(1, 431) = 4.95, *p*=0.026, and time, *F*(2, 633) = 157.82, *p*<0.001, such that participants in the CBT groups had reduced anxiety relative to the control group over the course of the follow-up periods. Baseline pain sensitivity significantly moderated the effect of the online CBT intervention on dental anxiety, *F*(1, 633) = 5.91, *p* = 0.015. Note: * = *p* < 0.05

To assess whether baseline pain sensitivity moderated the effect of the CBT intervention on dental anxiety across timepoints, a repeated-measures linear mixed-effects model was conducted. First, the model confirmed a direct effect of treatment condition on dental anxiety, such that participants who received the online CBT had a greater reduction in dental anxiety relative to the control group. The significant direct effect was observed between groups, *F*(1, 431) = 4.95, *p*=0.026, and across 1-month and 3-month follow-up timepoints, *F*(2, 633) = 157.82, *p*<0.001. A mixed-effects model with mean-centered interaction terms revealed a significant moderation effect of baseline pain sensitivity. Specifically, baseline pain sensitivity moderated the effect of the online CBT intervention on dental anxiety (Fig. 2), such that participants reporting lower pain sensitivity were significantly more likely to see reductions in dental anxiety when compared to those reporting higher pain sensitivity, *F*(1, 633) = 5.91, *p* = 0.015.

Figure 2 further illustrates the moderation effect, as the mean-centered pain sensitivity values were used to categorize participants into four groups based on intervention condition (CBT vs. control) and pain sensitivity level (high vs. low). Participants with low pain sensitivity who received the CBT intervention experienced the greatest reduction in mean dental anxiety from baseline to 1- and 3-month follow-ups (MDAS = 18.6, 15.5, and 14.2, respectively). Participants with low pain sensitivity who received the control condition experienced a similar pattern in dental anxiety reduction (MDAS = 18.9, 17.0, and 15.9, respectively), though at a slower rate than those who received the CBT intervention. Participants in the CBT intervention group with low baseline pain sensitivity scores saw the greatest improvement in dental anxiety – a 4.2-point reduction in total MDAS score from baseline to 3-month follow-up. Conversely, participants in the CBT intervention group with high baseline pain sensitivity experienced only a 3.1-point reduction in MDAS score. Among those who received the control condition, participants with low baseline pain sensitivity experienced a 3-point reduction and those with high pain sensitivity experienced a 2.5-point reduction in MDAS scores. Figure 2 highlights significant between groups differences at 1- and 3-month follow-ups (*p* < 0.05). Participants with high pain sensitivity who received the CBT intervention experienced a moderate, but consistent reduction in mean dental anxiety from baseline to 1- and 3-month follow-ups (MDAS = 20.7, 18.5, and 17.6, respectively). Meanwhile, participants with high baseline pain sensitivity who received the control intervention showed an initial reduction in dental anxiety at 1-month follow-up, followed by an uptick at 3-month follow-up (MDAS = 20.6, 17.2, and 18.1, respectively).

## Discussion

Cognitive behavioral therapy (CBT) is the standard-of-care treatment approach for anxiety disorders, including specific fears or phobias like dental anxiety (Gordon et al., 2013). CBT for specific phobias typically use an exposure and response prevention (ERP) intervention designed to allow patients to gradually face their feared stimuli while learning new coping strategies. There is a growing literature demonstrating the efficacy and effectiveness of CBT for dental anxiety, including computerized and web-based interventions (Tellez et al., 2015; Heyman et al., 2024). The current study adds to that literature by exploring patient-level factors that might predict the degree to which a patient responds (i.e., improves or benefits) from a brief online CBT program for dental anxiety. Specifically, this study demonstrates the important role of pain sensitivity among adults with dental anxiety, indicating that high pain sensitivity may inhibit treatment response. Adults with dental anxiety and low pain sensitivity benefited most from the brief online CBT intervention, exhibiting the greatest reduction in dental anxiety during the study observation period. Identifying key markers for treatment effects (e.g., pain sensitivity) can help with tailoring treatment recommendations based on individual characteristics.

This study found that adults with dental anxiety have different response patterns following an online CBT intervention based on their level of pain sensitivity. While those with lower pain sensitivity experienced a significant decrease in dental anxiety, individuals with higher pain sensitivity may require different or more intensive intervention. For instance, individuals with high pain sensitivity may have benefited from repeated exposures prior to advancing to the next level of the fear hierarchy. In practice, a patient would not advance to the next exposure stimuli until their subjective distress was sufficiently reduced (Abramowitz et al., 2019). The computerized program in this study advanced to the next exposure video regardless of the patients’ reported level of anxiety. Future research may consider addressing this question using a Sequential Multiple Assignment Randomized Trial (SMART) design (Collins et al., 2007), which could include a hierarchy of dental anxiety interventions ranging in duration (e.g., brief to prolonged) and intensity (e.g., computerized to individual therapy). Brief online CBT is an effective approach to managing dental anxiety, though the degree to which it is effective may depend of various individual-level factors.

This study had several strengths and limitations that should be considered. First, the study sample represents a diverse patient population with over 62% identifying as Black or African American and 11% as Hispanic/Latino, groups that are historically under-represented in clinical trials research. Minoritized communities bear the greatest burden of health inequity, access to care, and poor health outcomes. Recognizing the potential impact of dental anxiety among communities experiencing health disparities is particularly relevant, as lack of access to preventative care increases the risk of worse oral health outcomes and the likelihood of more invasive, expensive, and painful dental procedures. This pattern is likely to contribute, either as predisposing or perpetuating factors, to the cycle of fear and avoidance of dental care (Heyman et al., 2016; Seligman et al., 2017). As is common with randomized controlled trials, it is possible that the perception of being randomized to the active intervention or the control condition may have influenced the participant satisfaction with the research program or their responses on subsequent assessment of primary study outcomes. However, based on our analysis of data, we believe that this risk of bias is low. Participants in both groups reported high satisfaction with the programs. In fact, some participants in the control group perceived the non-dental videos (i.e., nature scenes) to be anxiety-reducing. Another limitation of the study is that participants were required to be patients of record at a dental school and have a scheduled appointment. This may limit the generalizability of this study, as the patient population may not be representative of adults who have dental homes at private dental practices. Additionally, adults with the most severe dental anxiety may not be represented, as they may avoid dental care altogether or cancel/no-show their appointments, and thus not have been identified throughout recruitment methods. To address these limitations, future research should explore implementation strategies that examine the brief online CBT intervention in community and private practice clinics and include efforts to enroll patients who avoid dental care altogether.
